# The Hypoglycemic Activity of *Gracilaria lemaneiformis* Polysaccharide Gels Based on IR/IRS-2/PI3k/Akt/Glut4 and Glycometabolism Signaling Pathways in HepG2 Cells

**DOI:** 10.3390/gels11050366

**Published:** 2025-05-15

**Authors:** Xiaoshan Long, Shucheng Liu, Xianqing Yang, Yongqiang Zhao, Shaoling Yang, Ya Wei, Chuang Pan, Shengjun Chen, Peihong Jiang, Bo Qi, Xiao Hu

**Affiliations:** 1 Key Laboratory of Aquatic Product Processing, Ministry of Agriculture and Rural, South China Sea Fisheries Research Institute, Chinese Academy of Fishery Sciences, Guangzhou 510300, China; longxs2023@163.com (X.L.);; 2 Guangdong Provincial Key Laboratory of Aquatic Product Processing and Safety, College of Food Science and Technology, Guangdong Ocean University, Zhanjiang 524088, China; 3 Key Laboratory of Efficient Utilization and Processing of Marine Fishery Resources of Hainan Province, Sanya Tropical Fisheries Research Institute, Sanya 572426, China; 4 Shandong Key Laboratory of Storage and Transportation Technology of Agricultural Products, Shandong Institute of Commerce and Technology, Jinan 250103, China

**Keywords:** polysaccharides gels, hypoglycemic mechanism, molecular weight, correlation analysis

## Abstract

The aim of this study was to investigate the hypoglycemic activity and mechanism of *G. lemaneiformis* polysaccharide gels (GLP and GLP-HV) based on IR/IRS-2/PI3k/Akt/Glut4 and glycometabolism signaling pathways in HepG2 cells. After H_2_O_2_-Vc degradation, the molecular weight of *G. lemaneiformis* polysaccharide gel declined from 1478 kDa to 16 kDa. Molecular weight chromatogram and distribution indicated that GLP-HV had a high molecular weight homogeneity compared to GLP. *G. lemaneiformis* polysaccharide gels significantly decreased the TC, TG, LDL-C, MDA, and LDH contents and enhanced the activities of HDL-C, T-AOC, CAT, GSH-PX, SOD, insulin, and glycogen in HepG2 cells. Fluorescent staining results showed that *G. lemaneiformis* polysaccharide gels reduced ROS and calcium ions levels in HepG2 cells. GLP and GLP-HV displayed excellent hypoglycemic activity, with GLP-HV performing better. Furthermore, qPCR and Western blot analysis revealed that *G. lemaneiformis* polysaccharide gels remarkably strengthened the levels of IR, IRS-2, PI3K, Akt, Glut4, HK, G6PD, PFK, PEPCK, GK, PK genes, and proteins. Spearman’s correlation analysis revealed that the IR/IRS-2/PI3k/Akt/Glut4 signaling pathway played a dominant role in regulating activity. These results show that *G. lemaneiformis* polysaccharide gels present a prominent hypoglycemic effect mediated by the IR/IRS-2/PI3k/Akt/Glut4 and glycometabolism signaling pathways, with the former playing a dominant role.

## 1. Introduction

Abnormal glucose metabolism, induced primarily by a high-fat and high-sugar diet, is accompanied by a range of diseases such as hyperlipidemia, diabetes, and cerebrovascular disease [1]. A high-fat and high-sugar diet may trigger insulin resistance, which disrupts the ability to regulate glucose, creating a glucose metabolism disorder [2]. Long-term abnormal glucose levels will damage organs and lead to the gradual decline of its function, which has become an important global public health problem [3]. At present, there are a large number of drugs to reduce blood glucose, but these drugs have toxic side effects on the human body and easily produce drug resistance. Thus, it is crucial to explore natural drug candidates with few side effects to regulate glucose metabolism disorders. Numerous studies have focused on the non-toxic side effects attributed to the active ingredients from plants and algae with hypoglycemic activity, including polysaccharides, polyphenols, tocopherols, and flavonoids [4,5,6,7]. It has been reported that *G. lemaneiformis* polysaccharide demonstrates high health and nutritional value, which can be further developed into health care products and functional foods [8,9,10].

The IR/IRS-2/PI3k/Akt/Glut4 signaling pathway plays an important role in hypoglycemic activity, promoting the regulation of glucose absorption and metabolism [11]. It has important effects on glucose and insulin homeostasis, improving insulin resistance, promoting gluconeogenesis, inhibiting glycolysis, and thus regulating glycolipid metabolism [12]. IR activates intracellular tyrosine kinase, promotes the phosphorylation of IRS-2, PI3K, and Akt, then stimulates the expression of glucose transporters (Glut2 and Glut4), which promotes gluconeogenic pathway, inhibits glycolysis, and regulates cellular glucose and lipid metabolism [13]. Polysaccharides have been found to up-regulate IR expression, promote IRS-2 phosphorylation, and stimulate PI3K expression [14]. The enhancement of IRS-2 may enhance insulin receptor sensitivity, which in turn enhances the effect of insulin, which regulates intracellular glucose homeostasis. Akt, a key node downstream of the PI3K/Akt signaling pathway, inhibits GSK-3*β* phosphorylation, promotes Glut4 expression, and regulates insulin and glycogen synthesis [15]. These relevant factors in the IR/IRS-2/PI3k/Akt/Glut4 signaling pathway participate in glucose and insulin homeostasis and interact with the glycometabolism signaling pathway.

Gluconeogenesis and glycolysis are the main pathways of glucose production and metabolism that exhibit excellent effects in regulating glucose stability. These glycometabolism signaling pathways provide metabolites against reactive oxygen species and oxidative stress, which can improve the oxidative damage of the organism and then improve glucose metabolism [16]. These changes may induce an enhancement in T-AOC, SOD, CAT, and GSH-PX levels and a decrease in MDA content, thus further improving the antioxidant capacity of the organism. Moreover, both gluconeogenesis and glycolysis have the function of regulating each other, and not only the inverse relation. PEPCK, G6PD, and PK belong to key rate-limiting enzyme in gluconeogenic pathway, and HK, GK, and PFK present the obvious function in glycolysis pathway. PEPCK is a key mediator of gluconeogenesis and plays a key role in the gluconeogenic metabolic pathway that converts oxaloacetic acid to pyruvate phosphate [17]. These rate-limiting enzymes perform crucial functions in the glycometabolism signaling pathways, reducing oxidative stress and regulating glucose metabolism. For example, PEPCK, a key mediator of gluconeogenesis, plays a crucial role in the uptake pathway that converts oxaloacetic acid to phosphodiesterate, which activates antioxidant production and relieves oxidative stress, thereby modulating glucose homeostasis metabolism [16,17]. HK is a key enzyme in the conversion of glucose into glucose-6-phosphatase, which effectively reduces glucose content [18]. The glycometabolism signaling pathway is closely related to the changes of glucose and glycogen content. Glucose and glycogen take part in this process that provides energy for cells and maintains glucose homeostasis. The synthesis of glycogen requires the consumption of glucose, which can be directly involved in the glycolysis process. The synthesis of glycogen requires the consumption of glucose in the process of gluconeogenesis, and the glucose produced by the breakdown of glycogen can be directly involved in the process of glycolysis. It is a crucial mechanism for regulating glucose formation and metabolism in the glycometabolism signaling pathway.

To further explore the hypoglycemic activity and its mechanism of *G. lemaneiformis* polysaccharide gels before and after degradation, the models of HepG2 cells with abnormal glucose metabolism were established. This study measured the biochemical indexes (TC, TG, HDL-C, LDL-C, T-AOC, SOD, MDA, CAT, GSH-PX, LDH, glycogen, insulin, ROS, and calcium ion) and probed the mRNA and proteins expression involved in IR/IRS-2/PI3k/Akt/Glut4 and glycometabolism signaling pathways in HepG2 cells. Moreover, Spearman’s correlation analysis of hypoglycemic activity and mechanism was investigated.

## 2. Results and Discussion

### 2.1. Molecular Weight Chromatogram and Distribution of G. lemaneiformis Polysaccharide Gels

**Figure 1:**
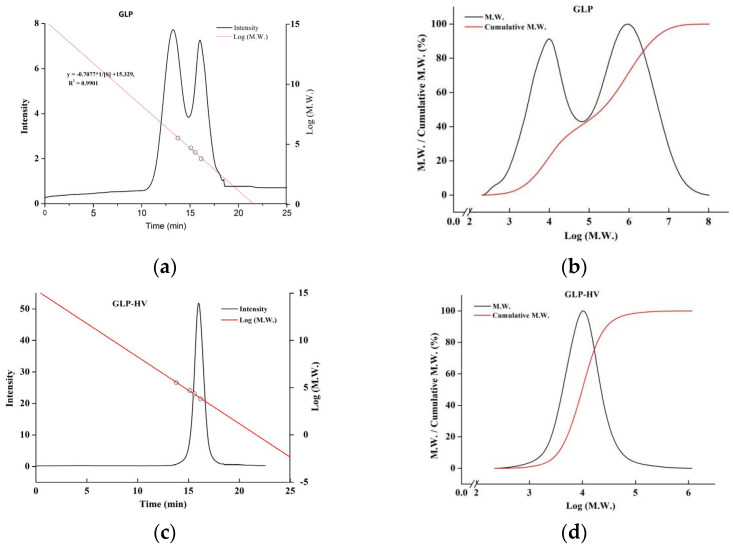
Molecular weight chromatogram and distribution of *G. lemaneiformis* polysaccharide gels. (**a**) Molecular weight chromatogram of GLP; (**b**) molecular weight distribution of GLP; (**c**) molecular weight chromatogram of GLP-HV; (**d**) molecular weight distribution of GLP-HV.

The molecular weight of GLP and GLP-HV were 1478 kDa and 16 kDa, respectively. H_2_O_2_-Vc caused the glyosidic bond to break and observably decreased the molecular weight of polysaccharide gels. As shown in Figure 1, GLP had two main components, while GLP-HV had only one main component and presented a narrower molecular weight distribution. The result showed that the degraded polysaccharide gel, GLP-HV, had high molecular weight homogeneity. The reduce of molecular weight may enhance the activity of polysaccharide gel. The H_2_O_2_-Vc degradation may break glycosidic bonds, reducing the molecular weight of *G. lemaneiformis* polysaccharide gels. Active groups may thus be exposed, increasing the hypoglycemic activity of polysaccharide gels. In our previous research, the polysaccharide gel degradation product showed better physiological activity in vitro [19].

### 2.2. Monosaccharide Composition of G. lemaneiformis Polysaccharide Gels

As shown in Table 1, the main monosaccharides of GLP and GLP-HV were glucose and galactose, with small amounts of mannose, ribose, glucuronide, galactose, arabinose, xylose, and fucose. GLP contained 34.35% glucose, 57.37% galactose, 0.274% galacturonic acid, and 0.187% glucuronic acid. GLP-HV contained 33.37% glucose, 59.12% galactose, 0.091% galacturonic acid, and 0.317% glucuronic acid, respectively. Degradation had little effect on the monosaccharides composition. However, galactose (92.85%) was the main monosaccharide from *G. lemaneiformis* polysaccharide gel studied by Tang et al. [20]. The main monosaccharides in the composition of *G. lemaneiformis* polysaccharide gel were galactose and anhydro-galactose, which was found by Zhang et al. [21]. Consistent with other studies, galactose was the main component.

The monosaccharides composition showed little change and was not affected by the breaking of glycosidic bonds. The possible reason was that the site where the glycosidic bond was broken could have been some other monosaccharides, or it could have been the site where the break occurred, exposing a new glucose or galactose group. All of these changes in chemical structure may induce an increase in hypoglycemic activity.

### 2.3. Cytotoxicity of *HepG2 Cells*

**Figure 2:**
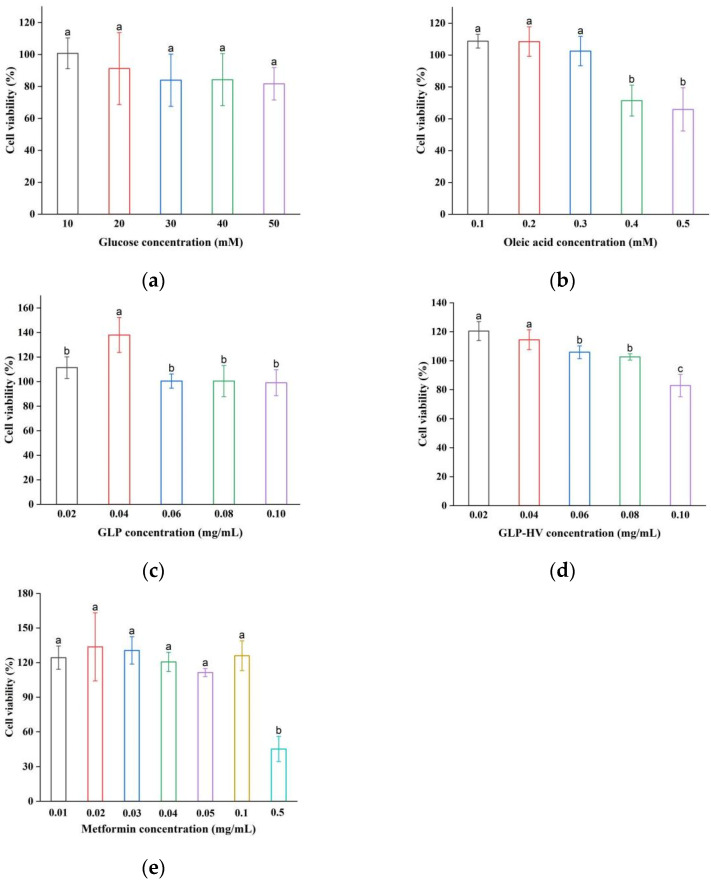
Cell viability of glucose, oleic acid, GLP, GLP-HV, and metformin in HepG2 cells. Note: (**a**) cell viability of glucose in HepG2 cells; (**b**) cell viability of oleic acid in HepG2 cells; (**c**) cell viability of GLP in HepG2 cells; (**d**) cell viability of GLP-HV in HepG2 cells; (**e**) cell viability of metformin in HepG2 cells. a, b and c represent significant differences, and *p* < 0.05 indicates significant differences.

The cell model of glycometabolism disorder was established by glucose and oleic acid, referring to the literature with minor modification [22]. Glucose and oleic acid normally affect the metabolism of HepG2 cells and induce insulin resistance, resulting in a state of high sugar and high lipid [23]. The toxicity of the cells increases with the samples content, which results in a decrease in cell viability. Moreover, different samples have different levels of inhibition on cell viability. Figure 2a,b shows that glucose had no toxic effect on cells in the range of 10–50 mM, while oleic acid inhibited cell growth at 0.4 mM. Chen et al. constructed a cell model with 0.2 mM oleic acid and found no toxic effects on the cells in this enrichment, in agreement with our experimental results [23]. Based on literature and cell viability tests, 10 mM glucose and 0.3 mM oleic acid were selected to build a HepG2 cell model with glycolipid metabolism disorders. Figure 2c,d show that GLP concentrations of 20–100 μg/mL had no toxic effect on HepG2 cells, and HepG2 cells’ viability was 82.87% ± 7.7 (still more than 80%) while GLP-HV concentration was 100 μg/mL. It was reported that the viability of HepG2 cells treated with blackberry polysaccharide at 50 μg/mL was 92.61% [24]. GLP and GLP-HV were relatively less toxic to cells compared to previous reports. Moreover, Metformin had no toxic effect on HepG2 cells at concentrations of 0.01–0.1 mg/mL, with a concentration of 0.05 mg/mL chosen as a positive control (Figure 2e). Therefore, GLP and GLP-HV with 20–100 μg/mL were selected to explore the regulation effect of glycometabolism on HepG2 cells.

### 2.4. The Hypoglycemic Activity and Mechanism of G. lemaneiformis Polysaccharide Gels in HepG2 Cells

#### 2.4.1. The Activities of TC, TG, HDL-C, LDL-C, T-AOC, SOD, MDA, CAT, GSH-PX, LDH, Glycogen, and Insulin

**Figure 3:**
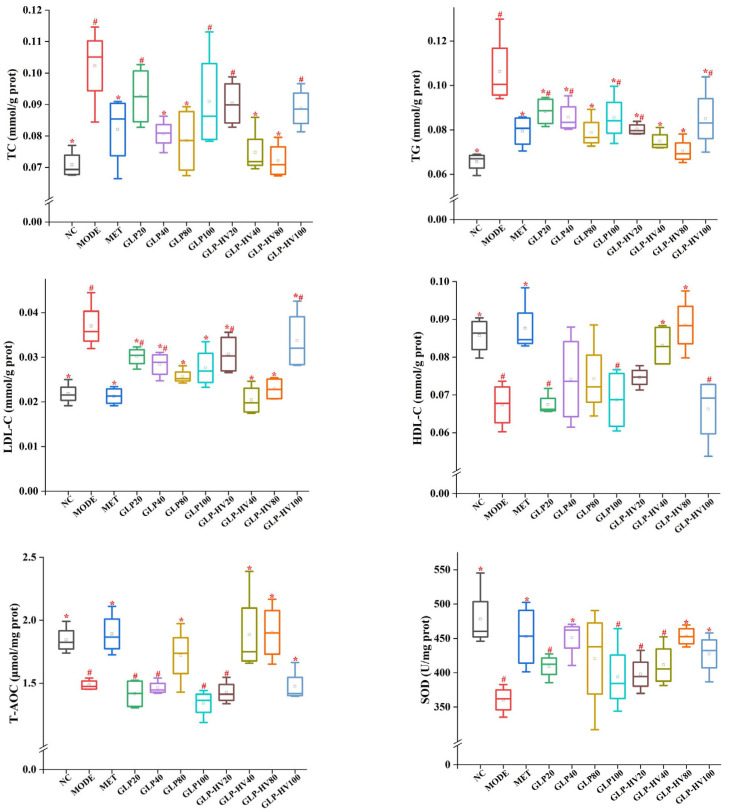

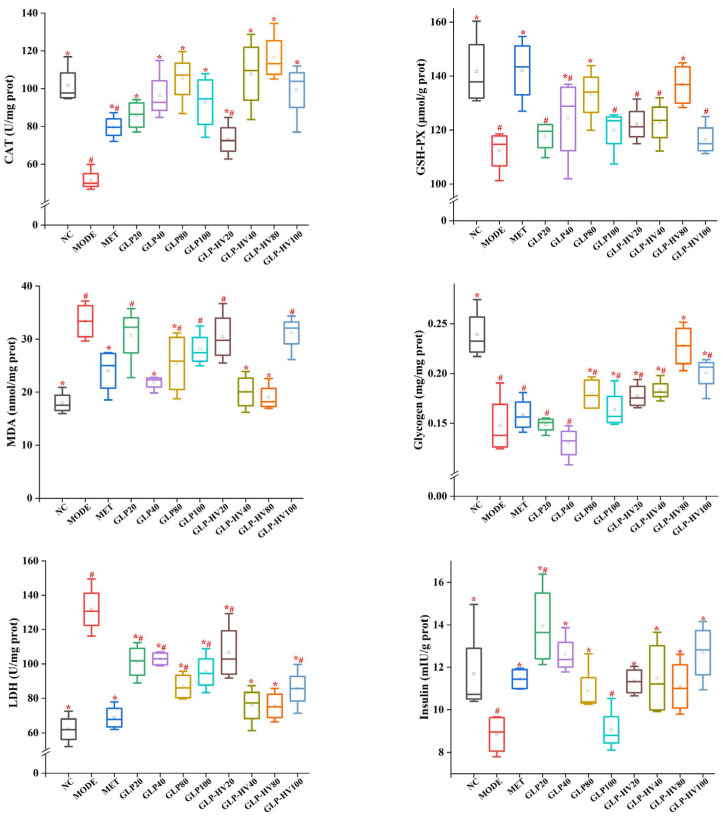
Hypoglycemic activity analysis of G. polysaccharide gels in HepG2 cells. Note: “*” represents significant differences between the other groups and MODE, “#” represents significant differences between the other groups and NC, and *p* < 0.05 indicates significant differences.

The activities of TC, TG, HDL-C, LDL-C, T-AOC, SOD, MDA, CAT, GSH-PX, LDH, glycogen, and insulin are displayed in Figure 3. The contents of TC, TG, and LDL-C were higher in MODE (0.1023 ± 0.0128 mmol/g prot, 0.1062 ± 0.0162 mmol/g prot, and 0.0370 ± 0.0053 mmol/g prot, respectively) than NC (0.0708 ± 0.0043 mmol/g prot, 0.0656 ± 0.0043 mmol/g prot, and 0.0218 ± 0.0024 mmol/g prot, respectively) (*p* < 0.05). Conversely, MODE markedly decreased HDL-C levels from 0.0857 ± 0.0047 mmol/g prot to 0.0674 ± 0.0059 mmol/g prot compared to NC. The results indicated that glucose and oleic acid caused abnormal glycometabolism. *G. lemaneiformis* polysaccharide gels supplement observably reduced the content of TC, TG, and LDL-C and enhanced HDL-C content, especially GLP-HV, which was in line with the literature [3].

Disorder in glucose metabolism promotes the production of large amounts of free radicals, which has a side effect on cell growth [8,25]. In a sense, T-AOC represents the total antioxidant capacity of the cells. The T-AOC level of GLP-HV was significantly stronger than that of MODE (*p* < 0.05) but not significantly different from that of NC (*p* > 0.05). Antioxidant enzymes such as SOD, CAT, and GSH-PX are effective in removing free radicals, increasing their scavenging capacity, and thus achieving glucose and fat reduction. The activities of SOD, CAT, and GSH-PX in NC were 477.96 ± 45.4 U/mg prot, 101.73 ± 10.33 U/mg prot, and 141.75 ± 13.49 μmol/g prot, respectively, which was higher than MODE (*p* < 0.05). GLP-HV had no significant difference from NC and presented excellent free radicals scavenging capacity (*p* > 0.05). MDA is a deleterious substance produced by peroxidation reactions in organisms which can cause cell damage and metabolic disorders and can reflect the degree of lipid peroxidation reactions in cells [26]. Compared with MODE, MDA contents in NC and MET were significantly decreased, and GLP-HV concentrations of 40 and 80 μg/mL were not significantly different from those in the normal group and positive drug group (*p* > 0.05), which effectively improved the free radicals scavenging capacity of cells.

Glycogen is a large molecule polysaccharide composed of glucose which is mainly stored in liver and skeletal muscle, namely liver glycogen and muscle glycogen [27]. Muscle glycogen is decomposed into muscle contraction to supply energy, and liver glycogen decomposition mainly maintains glucose concentration [28]. Compared with the NC group, the glycogen content in MODE was significantly decreased from 0.24 ± 0.03 mg/mg prot to 0.15 ± 0.03 mg/mg prot (*p* < 0.05). The marked reduction in glycogen content may be influenced by the activity of enzymes in the glycolysis and gluconeogenesis pathways, resulting in the breakdown of glycogen and an increase in intracellular glucose content. Both GLP and GLP-HV could improve the glycogen content, and there was no visible difference between GLP-HV with 80 μg/mL (glycogen content was 0.23 ± 0.002 mg/mg prot) and the NC group (*p* > 0.05). LDH is an enzyme involved in glycolysis and gluconeogenesis, and its activity has some effect on glycogen content and glucose concentration [29]. The LDH activity of MODE was significantly higher than that of the other groups, and GLP and GLP-HV exhibited a prominent effect on the recovery of LDH activity (*p* < 0.05). These results explained that glucose and oleic acid could lead to anomalous LDH activity, while *G. lemaneiformis* polysaccharide gels could prominently ameliorate LDH expression.

Insulin is an endogenous or exogenous substance received by the islet beta cells in the pancreas, which is the only hormone in the organism that can reduce glucose and promote the synthesis of glycogen, fat, and protein [30]. Insulin promotes glycometabolism, inhibits glycogen breakdown and gluconeogenesis, enhances glycolysis in muscle and fat, and promotes glucose entry into muscle and fat [22]. Furthermore, insulin inhibits or stimulates lipid metabolism-related enzymes (HSL), inhibiting lipolysis and reducing the supply of free fatty acids to the liver for ketogenic effects [31]. Abnormal glucose metabolism restrains glucose uptake and metabolic rate, resulting in insulin resistance and blocked binding to insulin receptors [22]. As shown in Figure 3, the insulin level of MODE was decreased compared to the NC group (8.85 ± 0.93 mIU/g prot vs. 11.70 ± 2.18 mIU/g prot, *p* < 0.05), and both GLP and GLP-HV showed observably enhanced insulin levels in the cells (*p* < 0.05). Sharma et al. confirmed that the insulin level of the mode group was less than that of the control group, which was similar to this research, though with the opposite conclusion [28]. Glucose and oleic acid disrupted the cell’s glycometabolism pathway, causing abnormal changes in the cell’s glycogen, LDH, and insulin levels. A lack of insulin will cause disordered fat metabolism and a reduction in fat storage, which can cause arteriosclerosis and lead to serious cardiovascular and cerebrovascular diseases. Insulin promotes uptake and utilization of glucose by systemic tissue cells and inhibits glycogen and gluconeogenesis breakdown [22]. GLP and GLP-HV were capable of repairing insulin resistance and ameliorating the cell’s glycogen and LDH levels. Therefore, insulin has the effect of lowering glucose. However, excess insulin level is able to induce a reduction in the number of insulin receptors, which can lead to insulin resistance. Glucose and oleic acid induced cells to produce free radicals, which cause some damage to the cells and thus increase their blood sugar and lipid levels. The immediate effects were changes in TC, TG, HDL-C, LDL-C, T-AOC, SOD, MDA, CAT, GSH-PX, LDH, glycogen, and insulin levels. Levels of cholesterol, free radicals, insulin, and glycogen showed a definite relation and expressed mutual effects.

The better performance of GLP-HV for hypoglycemic activity may be related to the molecular weight, relative viscosity, reduced sugar, sulfate group, carbon group, 3,6- anhydrogalactose, and the type and link position of the glycosidic bonds. In our previous research, GLP-HV had higher reduced sugar, sulfate group, carbon group, and 3,6-anhydrogalactose content and lower molecular weight and relative viscosity compared to GLP [19]. The total content of the monosaccharides composition did not change much, but the position of the monosaccharides may have changed. The physiological activity of GLP-HV displayed a positive relationship with 3,6-anhydrogalactose, reducing sugar, sulfate, and carbon groups level, and presented a negative effect with molecular weight and relative viscosity [19].

#### 2.4.2. ROS and Calcium Ions Analysis

**Figure 4:**
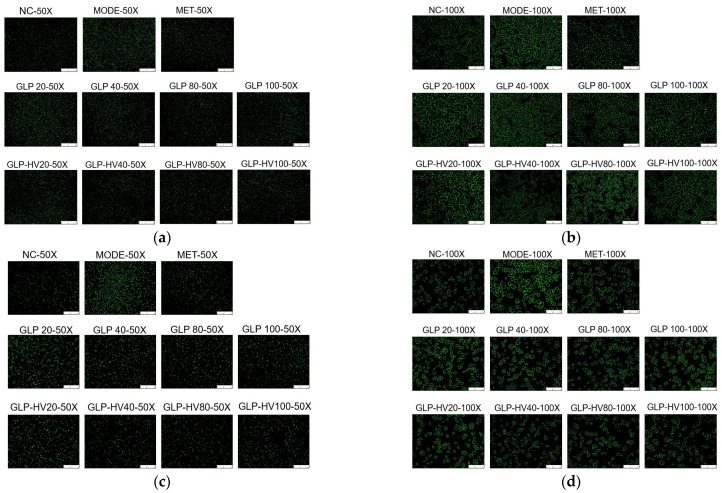
Fluorescent staining analysis of *G. lemaneiformis* polysaccharide gels in HepG2 cells. Note: (**a**) DCFH-DA fluorescent staining (50X) of GLP and GLP-HV; (**b**) DCFH-DA fluorescent staining (100X) of GLP and GLP-HV; (**c**) Fluo-3 AM fluorescent staining (50X) of GLP and GLP-HV; (**d**) Fluo-3 AM fluorescent staining (100X) of GLP and GLP-HV.

ROS is a by-product of organism metabolism, and excessive ROS will lead to oxidative damage of cells, which is an influencing factor for the disorder of cellular glycometabolism [12]. The DCFH fluorescent reagent is hydrolyzed by cellular esterase and then oxidized by peroxides into luminous substances, showing green fluorescence [32]. As displayed in Figure 4a,b, the green fluorescence intensity of MODE was the strongest, followed by the GLP and GLP-HV low-dose groups, and the GLP-HV with 40 and 80 μg/mL presented no significant difference compared with NC. GLP-HV presented better relief of oxidative stress effect compared to GLP and decreased the production of ROS. *G. lemaneiformis* polysaccharide gels effectively eliminated excessive ROS, alleviated oxidative stress, and enhanced the glucose metabolism capacity of cells. The result was consistent with TC, TG, HDL-C, LDL-C, T-AOC, SOD, MDA, CAT, GSH-PX, and other biochemical indices.

Calcium ions intensity reflects cell metabolism and oxidative damage, and high concentrations of calcium ions can also cause metabolic disorders and accelerate cell apoptosis [33]. Fluo-3 AM fluorescence probe is a common method to detect calcium ion concentration in cells, and it shows green fluorescence under an inverted fluorescence microscope [34]. As exhibited in Figure 4c,d, glucose and oleic acid induced cells to produce stronger calcium ion intensity, resulting in damage to certain cells, while GLP and GLP-HV, especially GLP-HV, had a certain repair ability on the injured cells, weakening the intracellular calcium ions concentration. GLP-HV with 40 μg/mL presented the best effect, that was similar to the ROS clear effect. In our previous studies, GLP-HV expressed better physiological activity of relieving oxidative stress in HepG2 cells induced by H_2_O_2_ [19]. The results proved that *G. lemaneiformis* polysaccharide gels provided an excellent effect on hypoglycemic activity.

These results indicated that glucose and oleic acid could promote oxidative damage and abnormal glucose metabolism of cells. GLP and GLP-HV, especially GLP-HV (40 and 80 μg/mL), could observably ameliorate oxidative damage and then regulated glucose and lipid metabolism in cells. In agreement with the above results, GLP-HV exhibited apparent hypoglycemic activity and regulated the biochemical indexes in HepG2 cells. After degradation, GLP-HV reduced the ROS and calcium ions contents and subsequently relieved oxidative stress in HepG2 cells. However, the dose of GLP-HV had a significant influence on the activity, with higher doses not being better. In this experiment, 40 and 80 μg/mL of GLP-HV showed the best effect on the control of ROS and calcium ions production.

### 2.5. The Regulation Mechanism of G. lemaneiformis Polysaccharide Gels Based on IR/IRS-2/PI3k/Akt/Glut4 and Gluconeogenesis Signaling Pathways in HepG2 Cells

#### 2.5.1. Gene Analysis in IR/IRS-2/PI3k/Akt/Glut4 and Gluconeogenesis Signaling Pathways

**Figure 5:**
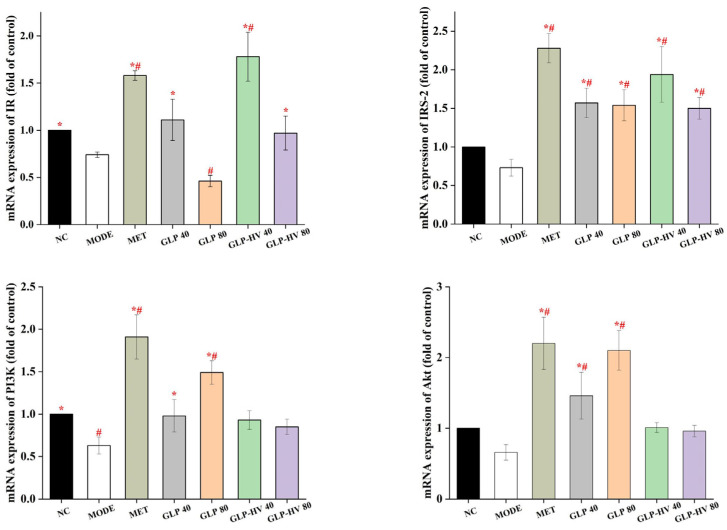

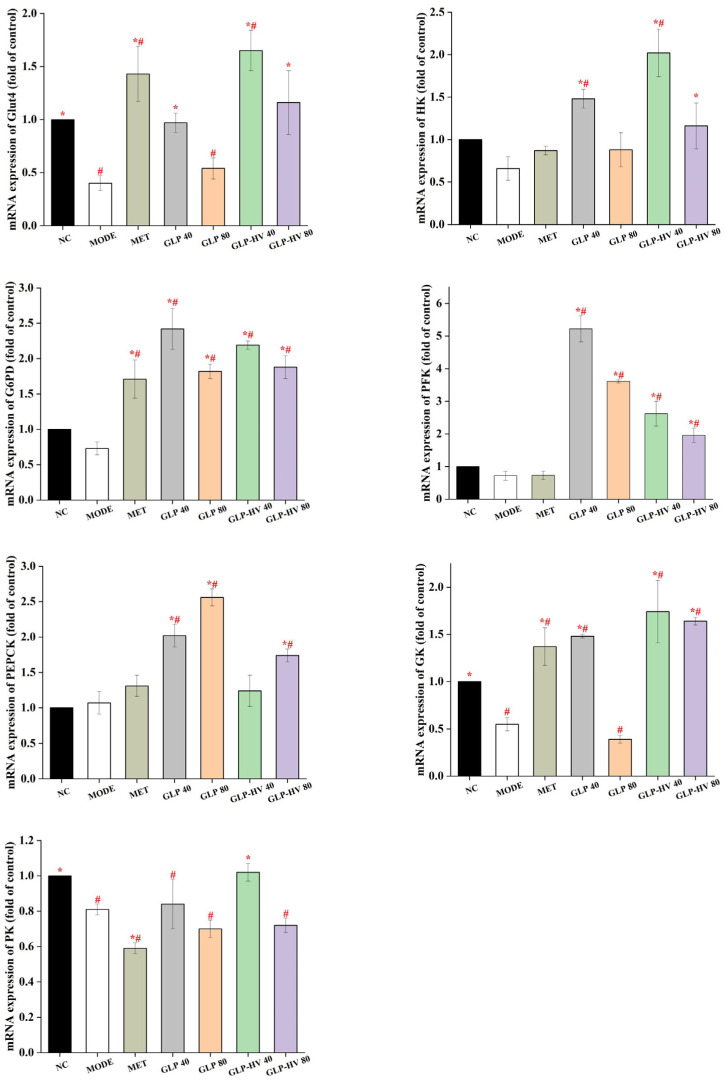
Gene analysis of the hypoglycemic signaling pathway of *G. lemaneiformis* polysaccharide gels in HepG2 cells. Note: “*” represents significant differences between the other groups and MODE, “#” represents significant differences between the other groups and NC, and *p* < 0.05 indicates significant differences.

The IR/IRS-2/PI3k/Akt/Glut4 signaling pathway plays an important role in the process of glucose metabolism and regulates the gluconeogenesis and glycolysis metabolic pathways, presenting a distinct hypoglycemic effect [14,35]. The *α*-subunit of IR can change the configuration of the *β*-subunit after binding with insulin, activate tyrosine protease, phosphorylate IRS, and then activate PI3K, so as to conduct intracellular signal conduction [36]. As revealed in Figure 5, mRNA expression of IR and IRS-2 were decreased in MODE compared to NC, and both GLP and GLP-HV up-regulated these two genes. The up-regulation of IR and IRS-2 triggered PI3K phosphorylation and stimulated an increase in PI3K gene expression, which in turn provoked mRNA expression of Akt and Glut4. Akt is a key node downstream of the PI3K/Akt signaling pathway, which activates upregulation of Glut4 [36]. Meanwhile, Glut4 is the primary glucose transporter in adipocytes and skeletal muscle cells and is essential for maintaining blood glucose balance in the organism [14]. *G. lemaneiformis* polysaccharide gels regulated genes expression in the IR/IRS-2/PI3k/Akt/Glut4 signaling pathway, particularly GLP-HV. The up-regulation of IR, IRS-2, PI3k, Akt, and Glut4 genes may promote the expression of HDL-C, T-AOC, SOD, CAT, GSH-PX, glycogen, and LDH and inhibit the production of TC, TG, LDL-C, MDA, ROS, and calcium ions. The hypoglycemic activity of *G. lemaneiformis* polysaccharide gels may be mediated by the IR/IRS-2/PI3k/Akt/Glut4 signaling pathway, which played an important regulatory role in insulin homeostasis by facilitating vesicle transport of Glut4, increasing the uptake and utilization of glucose. By inhibiting oxidative stress and improving mitochondrial respiratory function, *G. lemaneiformis* polysaccharide gels enhanced the energy metabolism capacity of the cell and modulated PEPCK, a key enzyme in gluconeogenesis and glycolysis.

HK, G6PD, PFK, PEPCK, GK, and PK belong to the key enzymes involved in glycometabolism and play an important role in glucose stability and metabolism [28]. As shown in Figure 5, mRNA expression of HK, G6PD, PFK, and PEPCK had no significant difference between NC and MODE, whereas GLP and GLP-HV enhanced expression of these genes. HK is a key enzyme in the conversion of glucose into glucose-6-phosphatase, which effectively reduces glucose content [18]. The mRNA level of HK of GLP-HV with 40 μg/mL was more than 2 times that of the MODE group (*p* < 0.05). PEPCK is a key mediator of gluconeogenesis, playing a key role in the uptake pathway that converts oxaloacetic acid to pyruvate phosphate and activates antioxidant production, which in turn modulates glucose homeostasis metabolism [16]. In this experiment, GLP-HV (40 μg/mL) had no prominent discrepancy compared to the MODE group, and GLP-HV (80 μg/mL) showed an obvious effect. Moreover, mRNA expression of GK and PK in MODE group were markedly down-regulated compared to NC, and GLP-HV (40 μg/mL) observably up-regulated these two genes (*p* < 0.05). Based on the above results, GLP-HV expressed a remarkable effect on glycometabolism. *G. lemaneiformis* polysaccharide gels regulated gluconeogenesis and glycolysis enzymes, inhibited lipid decomposition in cells, promoted glucose utilization, reduced free fatty acid accumulation, thereby improving lipid aggregation in cells, and had a regulatory effect on cellular glycometabolism.

#### 2.5.2. Proteins Expression in IR/IRS-2/PI3k/Akt/Glut4 and Gluconeogenesis Signaling Pathways

**Figure 6:**
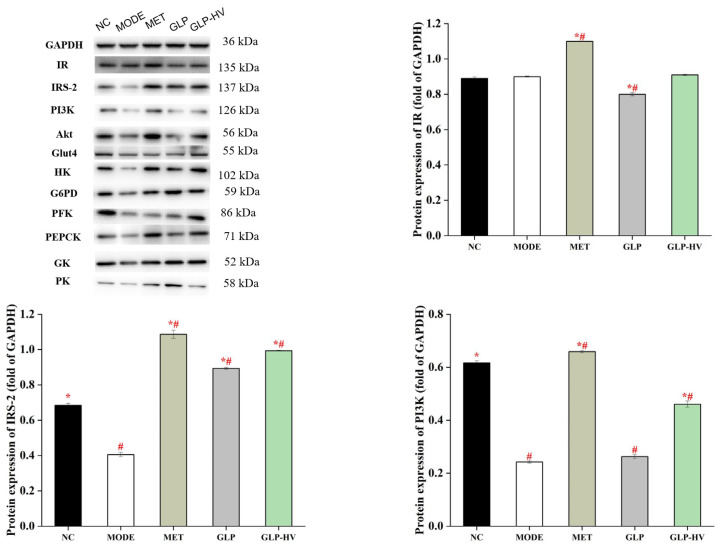

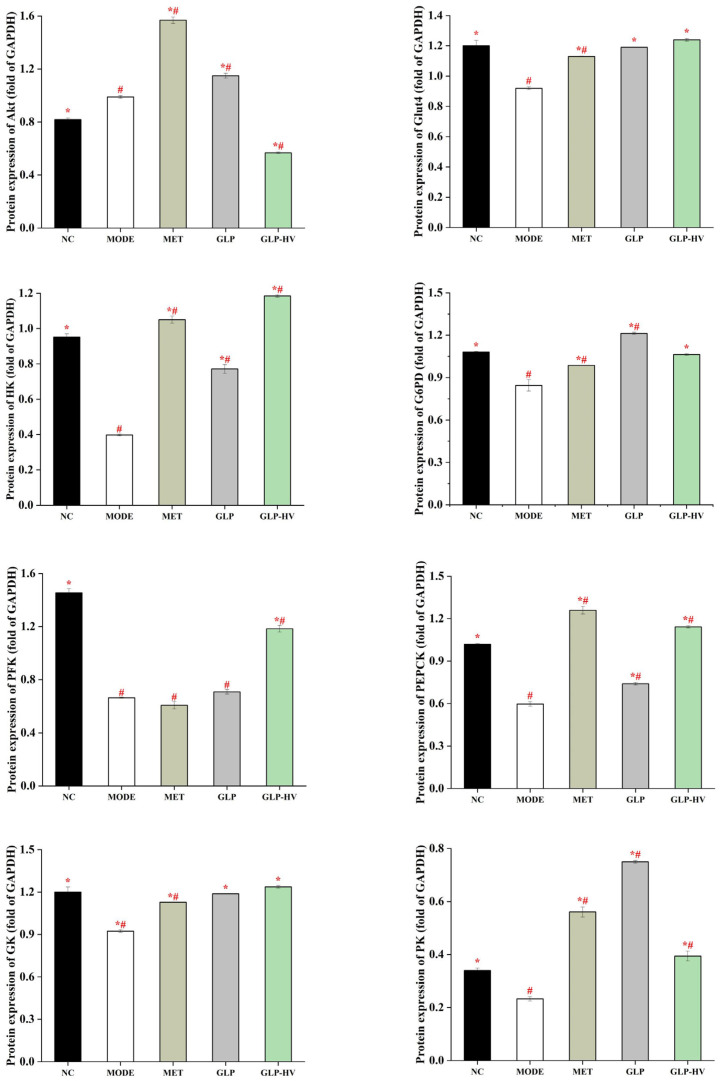
Western blot analysis of the hypoglycemic signaling pathway of *G. lemaneiformis* polysaccharide gels in HepG2 cells. Note: “*” represents significant differences between the other groups and MODE, “#” represents significant differences between the other groups and NC, and *p* < 0.05 indicates significant differences.

To further disclose the hypoglycemic mechanism of *G. lemaneiformis* polysaccharide gels, the proteins expression of IR/IRS-2/PI3k/Akt/Glut4 and glycometabolism signaling pathways are exhibited in Figure 6. The proteins expression of IRS-2, PI3k, and Glut4 were reduced in MODE, and GLP-HV treatment remarkably promoted these proteins’ levels (*p* < 0.05). However, there was no significant difference in IR protein expression between NC, MODE, and GLP-HV (*p* > 0.05). GLP improved the Akt protein level, while GLP-HV down-regulated it. IRS-2 is involved in the phosphorylated protein of insulin and other cytokine signaling. The apparent enhancement of IR-2 can bind to PI3K, stimulating its activity and accelerating phospholipid metabolism. Apparently, GLP-HV advanced the PI3K protein, promoting the expression of Glut4. Phosphorylation and protein expression of relevant factors in the IR/IRS-2/PI3k/Akt/Glut4 signaling pathway maintain glucose and insulin metabolism and homeostasis.

In addition, PEPCK, G6PD, and PK are key rate-limiting enzymes in the gluconeogenic pathway, and HK, GK, and PFK present distinct functions in the glycolytic pathway. These key rate-limiting enzymes participate in the crucial process in glycometabolism signaling pathways that activate antioxidant production and relieve oxidative stress, thereby modulating glucose homeostasis metabolism [16,17]. GLP and GLP-HV treatment, especially GLP-HV, up-regulated the expression of HK, G6PD, PFK, PEPCK, GK, and PK proteins, which played a positive role in gluconeogenesis and glycolysis, promoting glucose metabolism and contributing to homeostasis in the internal environment. The enhancement of these key enzymes in the glycometabolism signaling pathways promotes the activity of T-AOC, SOD, CAT, and GSH-PX and decreases the MDA content, thereby reducing oxidative stress. Alternatively, the enhanced glycogen content can be attributed to the enhancement of the rate-limiting enzymes in the gluconeogenic pathway, which facilitates the process of glycogen synthesis.

### 2.6. Correlation Analysis of the Activity and Mechanism of Hypoglycemic Mechanism by G. lemaneiformis Polysaccharide Gels in HepG2 Cells

**Figure 7:**
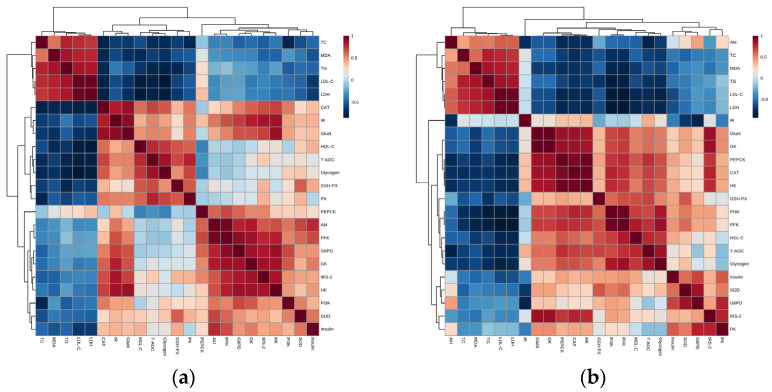
Spearman’s correlation between hypoglycemic activity and mechanism of *G. lemaneiformis* polysaccharide gels. Note: (**a**) the correlation between biochemical indexes and genes of signaling pathways; (**b**) the correlation between biochemical indexes and proteins of signaling pathways.

Figure 7a,b present the correlation between hypoglycemic activity and the mechanism of *G. lemaneiformis* polysaccharide gels. The heat map in Figure 7a indicates that TC, TG, LDL-C, MDA, and LDH activities were negatively correlated with the genes in both signaling pathways, except for PEPCK. Conversely, the correlation between CAT and genes was completely positive, except for PEPCK. Insulin was shown to have a weak correlation with the PK gene and a positive correlation with other genes in the signaling pathways. In particular, Akt and PFK showed a clear effect on insulin levels, improving insulin resistance. HDL-C, T-AOC, GSH-PX, and glycogen showed a strong positive correlation with IR, Glut4, and PK genes and a negative correlation with PEPCK. In addition, T-AOC and GSH-PX activities showed the slight positive correlation with IRS-2 and PI3K genes, and glycogen had a positive relationship with PI3K gene. The IR/IRS-2/PI3k/Akt/Glut4 and glycometabolism signaling pathways showed a significant reduction in oxidative stress followed by an improvement in glucose metabolism.

As shown in Figure 7b, TC, TG, LDL-C, MDA, and LDH activities were negatively correlated with these proteins in the signaling pathway, while a positive relation with Akt emerged. However, the correlation between CAT and proteins in the signaling pathway is contrary to the results presented above. Moreover, insulin, SOD, and GSH-PX displayed a positive relationship with proteins in the signaling pathways, except for IR and Akt. Furthermore, the up-regulation of IRS-2, PI3K, Glut4, HK, PFK, PEPCK, and GK proteins promoted the activity of HDL-C, T-AOC, and glycogen. Consequently, these results revealed that the IR/IRS-2/PI3k/Akt/Glut4 and glycometabolism signaling pathways intensified the activity of HDL-C, T-AOC, CAT, GSH-PX, SOD, insulin, and glycogen and suppressed the content of MDA TC, TG, LDL-C, MDA, and LDH. Generally speaking, the IR/IRS-2/PI3k/Akt/Glut4 signal pathway played a dominant role.

## 3. Conclusions

After H_2_O_2_-Vc degradation, the molecular weight of *G. lemaneiformis* polysaccharide gels was decreased from 1478 kDa to 16 kDa. Molecular weight chromatogram and distribution indicated that GLP-HV had a high molecular weight homogeneity compared to GLP. *G. lemaneiformis* polysaccharide gels have been observed to reduce TC, TG, and LDL-C and increase HDL-C contents in HepG2 cells, improving the glycometabolism disorders induced by high glucose and oleic acid. In addition, *G. lemaneiformis* polysaccharide gels distinctly enhanced T-AOC, SOD, CAT, and GSH-PX levels and decreased the MDA level. Fluorescence results revealed that ROS and calcium ions intensity were weakened after treatment with *G. lemaneiformis* polysaccharide gels, which alleviated the oxidative damage and fortified the glycometabolism process. Moreover, *G. lemaneiformis* polysaccharide gels augmented glycogen and insulin levels, weakened the LDH level, and improved insulin resistance in HepG2 cells. GLP and GLP-HV displayed excellent hypoglycemic activity, with GLP-HV performing better. Furthermore, the expression of genes and proteins involved in the IR/IRS-2/PI3k/Akt/Glut4 and glycometabolism signaling pathways were up-regulated or down-regulated via *G. lemaneiformis* polysaccharide gels. Spearman’s correlation confirmed that these two signaling pathways intensified the activities of HDL-C, T-AOC, CAT, GSH-PX, SOD, insulin, and glycogen and suppressed the content of TC, TG, LDL-C, MDA, and LDH, thus heightening the hypoglycemic effect of *G. lemaneiformis* polysaccharide gels, with the IR/IRS-2/PI3k/Akt/Glut4 signaling pathway playing a dominant role. Currently, our experiments have shown that *G. lemaneiformis* polysaccharide gels present activity in regulating hyperglycemia in HepG2 cells. Further verification through animal experiments is still needed in the future.

## 4. Materials and Methods

### 4.1. Materials and Chemicals

*G. lemaneiformis* was purchased from Nan’ao Island (Shantou, Guangdong, China); T-AOC ELISA kit was bought from ZCIBIO Technology Co., Ltd. (Shanghai, China); RIPA, Fluo-3 AM, and CCK-8 were purchased from Solarbio Biotechnology Co., Ltd. (Beijing, China); BCA, SOD, MDA, CAT, GSH-PX, insulin, and LDH assay kit were purchased from Nanjing Jiancheng Biotechnology Co., Ltd. (Nanjing, China); glucose oxidase kit was purchased from Beijing Applygen Technology Co., Ltd. (Beijing, China); glycogen kit was purchased from Beijing Box Biotechnology Co., Ltd. (Beijing, China); 2′,7′-dichlorofluorescein diacetate (DCFH-DA) was purchased from Shanghai Yuanye Biotechnology Co., Ltd. (Shanghai, China).

### 4.2. Preparation of G. lemaneiformis Polysaccharide Gels

The extraction and degradation methods of *G. lemaneiformis* polysaccharide gels were described in previous studies [19]. The polysaccharide gel of *G. lemaneiformis* was extracted by hot water and ethanol precipitation and named GLP. GLP was degraded by vitamin C and H_2_O_2_ (H_2_O_2_-Vc), which was named GLP-HV.

### 4.3. Determination of Molecular Weight

The molecular weight of polysaccharide gels were determined by gel permeation chromatography (GPC) [37]. The specific parameters were as follows: TSKgel GMPWXL aqueous gel column (TOSOH Company, Tsushima, Japan), mobile phase consisted of 0.1 M NaNO_3_ and 0.06% NaN_3_ aqueous solution, flow rate was 0.6 mL/min, column temperature was 35 °C.

### 4.4. Determination of Monosaccharide Composition

Monosaccharide composition was determined using high-performance liquid chromatography (LC-20AD) according to the method reported by Kang et al. [38].

### 4.5. Cell Culture

HepG2 cells (CX0004), which were purchased from the Boster Biological Technology Co., Ltd. (Wuhan, China), were cultured with MEM (Minimum Essential Medium) medium (containing 10% FBS and 1% penicillin-streptomycin) and placed in an incubator with 5% CO_2_ at 37 °C. When the adherent growth area of the cells reached 80%, they were subcultured. In the fifth generation, drugs were added, and the medium was changed every two days during this period [23].

### 4.6. Cell Viability Assay

The effects of glucose, oleic acid, GLP, GLP-HV, and metformin on the viability of HepG2 cells were determined by the CCK-8 kit, which was referred to the previous research [19]. Cell viability was calculated by the following formula:

Cell viability (%) = (A − A_blank_)/(A_0_ − A_blank_) × 100 (1)

Note: A: absorbance values of cells, CCk-8, glucose, oleic acid, GLP, GLP-HV, and metformin; A_blank_: absorbance values of medium and CCk8; A_0_: absorbance values of cells and CCk-8.

### 4.7. Establishment of HepG2 Cells Model with Hyperglycemia

The cell model was built using glucose (10 mM) and oleic acid (0.3 mM), with reference to the results of cell viability assay and literature improvements [22,39]. The normal group (NC), model group (MODE), positive drug group (MET, metformin concentration was 0.05 mg/mL), GLP group (GLP 20, 40, 80 and 100 μg/mL), and GLP-HV group (GLP-HV 20, 40, 80 and 100 μg/mL) were set up. Metformin, GLP, and GLP-HV were prepared with sterile water, filtered with a 0.22 μm filter membrane, and diluted to the corresponding concentration for the experiment.

### 4.8. Biochemical Indexes

The activities of TC, TG, HDL-C, LDL-C, T-AOC, SOD, MDA, CAT, GSH-PX, LDH, glycogen, and insulin were referred to the literature with modification [40], which was operated according to the kit instructions. The protein content of HepG2 cells was detected by the BCA assay kit.

### 4.9. ROS Intensity

The intensity of ROS in HepG2 cells was studied by 2′, 7′-dichlorofluorescein diacetate (DCFH-DA) via an inverted fluorescence microscope (Leica Application Suite X 3.4.2.18368, Shanghai, China) without light [37].

### 4.10. Calcium Ions Intensity

Calcium ions intensity in HepG2 cells was studied by Fluo-3 AM via an inverted fluorescence microscope [19].

### 4.11. Quantitative Real-Time (qRT-PCR)

qRT-PCR detection was referred to the literature with modification [19]. In this study, the mRNA of target genes were searched on NCBI, and primers were designed and synthesized. The primers used for the RT-PCR assay are presented in the Supplementary Material (Table S1. Primers used for RT-PCR assay). The cells were washed twice with PBS and lysed with Trizol reagent. RNA was extracted from the cells and then reverse-transcribed into cDNA, which was amplified by fluorescence quantitative PCR.

### 4.12. Western Blot

Protein was extracted by pre-cooled TPEB (Total Protein Extraction Buffer), and protein concentration was determined with the BCA kit. SDS-PAGE gel (10% SDS-PAGE separation glue and 5% SDS-PAGE concentrate glue) was placed into the electrophoresis tank and the sample was added; SDS-PAGE gel at the end of electrophoresis was transcoated (semi-dry); antibodies were added for immunohybridization and color rendering [41].

### 4.13. Statistical Analysis

Data are expressed as means ± standard (SD). Duncan’s multiple range test was applied to identify differences between the mean values for each group by IBM SPSS software (version 22). *p* < 0.05 was considered to represent statistical significance. Figures were finished by Origin 8.5.

## Figures and Tables

**Table 1:** Monosaccharides composition of GLP and GLP-HV.

Monosaccharide (%)	GLP	GLP-HV
Glucose	34.354	33.372
Galactose	57.367	59.123
Mannose	0.189	0.272
Ribose	0.019	0.659
Rhamnose	1.329	0.363
Xylose	0.123	0.614
Arabinose	1.085	0.748
Fucose	5.074	4.441
Glucuronic acid	0.187	0.317
Galacturonic acid	0.274	0.091

## Data Availability

All data are included in the manuscript.

## Supplementary Materials

The following supporting information can be downloaded at: https://www.mdpi.com/article/10.3390/gels11050366/s1, Table S1. Primers used for RT-PCR assay. Table S2: Chemical shifts of resonances in the 1H and 13C spectra of GLP-HV.

## Abbreviations

The following abbreviations are used in this manuscript:

HepG2	Human hepatocellular carcinomas
TC	Total cholesterol
TG	Triglyceride
HDL-C	High-density lipoprotein cholesterol
LDL-C	Low-density lipoprotein cholesterol
T-AOC	Total antioxidant capacity
SOD	Superoxide dismutase
MDA	Malondialdehyde
CAT	Catalase
GSH-PX	Reduced glutathione hormone
LDH	Lactate dehydrogenase
IR	Insulin resistance
IRS-2	Insulin receptor substrate 2
PI3k	Phosphatidylinositol3-kinase
Akt	Protein kinase B
Glut4	Glucose transporter 4
HK	Gluconeogenic pathway, and hexokinase
G6PD	glucose-6-phosphatase
PFK	Phosphofructokinase
PEPCK	Phosphoenolpyruvate carboxykinase
GK	Glucokinase
PK	Pyruvate kinase
