# Supplementary material for: The Hypoglycemic Activity of Gracilaria lemaneiformis Polysaccharide Gels Based on IR/IRS-2/PI3k/Akt/Glut4 and Glycometabolism Signaling Pathways in HepG2 Cells
Source: Gels. 2025 May 15;11(5):366. doi: 10.3390/gels11050366 (PMC12110834; doi:10.3390/gels11050366)
Supplement: Supplementary file 1 [file gels-11-00366-s001.zip › gels-3587109-supplementary.pdf]

Table S1. Primers used for RT-PCR assay

Abbreviation	Full name	Forward（5’-3’）	Reverse
IR	Insulin Resistance	CACCTTGGTGAGCTCTGGAC	GACAGTCCTGGGAAGAGCAC
IRS-2	insulin receptor substrate 2	GCTGCTGCTACAGCTCCT	GGCTCGCCAAAGTCGATGT
PI3k	phosphatidylinositol3-kinase	CCAAATGAAAAGAACGGCTA	GCGACTTCAGCTTATCATGG
Akt	protein kinase B	GAGCATCATCCCTGGGTTAC	CTCCTTCACAATGGCTACG
Glut4	glucose transporter 4	ACCATAGGAGCTGGTGTGGTCAAT	GACCCATAGCATCCGCAACA
HK	hexokinase	ACGGAGCTGAAGGATGACCAG	CCCTTTTCAGAGCCATCAGGAATG
G6PD	glucose-6-phosphatase	AGGAAGGATGGAGGAAGGAA	TGGAACCAGATGGGAAAGAG
PFK	Phosphofructokinase	TTGGAACCACCTTGACCAGTCC	CTGTTCGCTCTACCGTGAGGAT
PEPCK	phosphoenolpyruvate carboxykinase	GTTCCCAGGGTGCATGAAAG	AGGGCGAGTCTGTCAGTTCAA
GK	glucokinase	GTGTACAAGCTGCACCCGA	CAGCATGCAAGCCTTCTTG
PK	pyruvate kinase	CAGAGAAGGTCTTCCTGGCTCA	GCCACATCACTGCCTTCAGCAC
GAPDH	glyceraldehyde-3-phosphate dehydrogenase	GGAGAAACCTGCCAAGTATGATGAC	GAGACAACCTGGTCCTCAGTGTA

Table S2. Chemical shifts of resonances in the ^1H and ^{13}C spectra of GLP-HV

Code	Glycosyl residues	Chemical shifts(ppm)					
		H1/C1	H2/C2	H3/C3	H4/C4	H5/C5	H6a,6b/C6
A	→4)-*α*-D-Glc*p*-(1→	5.05	4.05	4.45	3.54	3.86	3.64,3.85
		97.94	68.47	79.6	76.79	73.23	60.88
B	→3)-*β*-D-Gal*p*-(1→	4.47	4.02	3.69	3.52	3.49	3.74,3.59
		101.83	71.34	81.65	71.49	74.94	60.41
C	*β*-D-Glc*p*-(1→	4.35	3.6	3.87	3.71	3.61	3.64,3.85
		102.87	72.69	71.67	71.15	72.79	60.88
D	→4,6)-*α*-D-(2-O-Me)-Glc*p*-(1→	5.3	3.54	3.75	3.52	not detected	4.28,3.93
		99.62	71.42	71.17	81.54	not detected	69.17
